# Manganese-free chow, a refined non-invasive solution to reduce gastrointestinal signal for T_1_-weighted magnetic resonance imaging of the mouse abdomen

**DOI:** 10.1177/0023677219869363

**Published:** 2019-09-16

**Authors:** Veerle Kersemans, Sheena Wallington, Philip D Allen, Stuart Gilchrist, Paul Kinchesh, Richard Browning, Katherine A Vallis, Kathrin Schilling, Phil Holdship, Lee-Anne Stork, Sean Smart

**Affiliations:** 1 Cancer Research UK/MRC Oxford Institute for Radiation Oncology, Department of Oncology, University of Oxford, Oxford, UK; 2 Department of Earth Sciences, University of Oxford, Oxford, UK

**Keywords:** ethics and welfare, gastrointestinal hyperintensities, manganese, mouse, MRI, refinement

## Abstract

Commercial mouse chow is designed to provide a complete, nutrient-rich diet, and it can contain upwards of 100 mg/kg manganese, an essential mineral. Manganese acts as a relaxation time-shortening contrast agent for both T_1_ and T_2_, and where standard chow is hydrated in the gastrointestinal tract, bright signals are produced when using T_1_-weighted imaging (T_1_WI). As a result of peristalsis, gastrointestinal hyperintensities result in temporally unstable signals, leading to image ghosting and decreased resolution from that prescribed. To avoid the problem, various methods of gastrointestinal tract modulation, including the use of intestinal cleansing with laxatives and dietary modulation, have been reported. Here, dietary modulation has been extended to the use of a biologically innocuous, long-term change of diet. In this study, we report on the use of a commercially available manganese-free chow to improve the image quality of the gastrointestinal tract. This manganese-free chow, apart from the omitted manganese which is available in tap water, is a complete diet and readily available. We investigated the time-dependent, diet-related gastrointestinal intensities on short-TR T_1_WI magnetic resonance imaging; monitored body mass, food and water consumption and standard blood biochemistry analysis following diet change; and determined manganese concentration in blood plasma following a five-day change to manganese-free chow. We show that the manganese-free chow presents a refinement to other gastrointestinal tract modulation, as it avoids the need for invasive procedures for gut voiding and can be provided ad libitum so that animals can be maintained with no need for prescribed diet change before imaging.

## Résumé

L’alimentation commerciale des souris est conçue pour fournir une alimentation riche en nutriments et peut contenir jusqu’à 100 mg/kg manganèse, un minéral essentiel. Le manganèse agit comme un agent de contraste raccourcissant le temps de relaxation de T1 et de T2, et lorsque l’alimentation standard est hydratée dans le tractus gastro-intestinal, des signaux lumineux sont produits lors des examens d’IRM pondérée en T1 (T1WI) En tant de conséquence de la péristase, les hyperintensités gastro-intestinales découlent de signaux temporairement instables qui entraînent des dédoublements d’images et une résolution de moindre qualité par rapport à ce qui est prescrit. Pour éviter le problème, différentes méthodes de modulation du tractus gastro-intestinal ont été rapportées, dont l’utilisation de laxatifs pour nettoyer l’intestin et la modulation alimentaire. La modulation alimentaire a été étendue ici à l’adoption d’un changement de régime alimentaire biologiquement anodin et à long terme. Dans cette étude, nous rapportons l’utilisation d’une alimentation sans manganèse disponible dans le commerce pour améliorer la qualité des images du tractus gastro-intestinal. L’alimentation sans manganèse est une alimentation complète disponible dans le commerce, mais sans manganèse, lequel se trouve dans l’eau du robinet. Nous avons examiné les intensités gastro-intestinales dépendantes du temps et liées au régime alimentaire lors d’IRM en T1WI avec un TR court; la consommation d’eau et d’aliments et l’analyse biochimique standard du sang après un changement de régime alimentaire; et déterminé la concentration plasmatique de manganèse dans le sang après cinq jours de régime alimentaire sans manganèse. Nous avons montré que l’alimentation sans manganèse permettait un raffinement d’autres modulations du tractus gastro-intestinal car elle évitait d’avoir besoin de procédures invasives pour vider l’intestin et était disponible en permanence, les animaux pouvant donc être conservés sans avoir besoin de leur prescrire un changement de régime alimentaire avant l’imagerie.

## Abstract (de)

Kommerzielles Mäusefutter soll eine vollwertige, nährstoffreiche Ernährung gewährleisten und kann den lebenswichtigen Mineralstoff Mangan in einer Konzentration von bis zu 100 mg/kg enthalten. Mangan wirkt als die Erholungszeit verkürzendes Kontrastmittel für T1 und T2, und wenn Standard-Futter im Magen-Darm-Trakt hydratisiert wird, werden mit der T1-gewichteten Tomografie (T1WI) helle Signale erzeugt. Infolge der Peristaltik verursachen gastrointestinale Hyperintensitäten temporär instabile Signale, was zu Geisterbildern und einer Auflösung führt, die unter der vorgeschriebenen liegt. Zur Vermeidung des Problems wurden verschiedene Methoden der Gastrointestinaltraktmodulation beschrieben, darunter die Darmreinigung mit Abführmitteln und die diätetische Modulation. In der vorliegenden Studie wurde die diätetische Modulation durch die Nutzung einer biologisch unbedenklichen, langfristigen Ernährungsumstellung erweitert. Wir berichten hier über die Verwendung eines handelsüblichen manganfreien Futters zur Verbesserung der Bildqualität des Magen-Darm-Traktes. Dieses manganfreie Futter ist, abgesehen vom Wegfall von Mangan, das im Leitungswasser enthalten ist, eine vollwertige und leicht verfügbare Nahrung. Wir untersuchten die zeitabhängigen, ernährungsbedingten gastrointestinalen Intensitäten bei kurzer TR, T1WI MRT, überwachten Körpergewicht, Nahrungsmittel- und Wasserverbrauch und standardmäßige biochemische Blutanalysen nach Ernährungsumstellung und bestimmten Mangankonzentrationen im Blutplasma nach einer 5-tägigen Umstellung auf manganfreies Futter. Wir zeigen, dass das manganfreie Futter eine Verbesserung gegenüber anderen Modulationen des *Gastrointestinaltrakts* darstellt, weil es invasive Verfahren zur Darmentleerung überflüssig macht und nach Belieben bereitgestellt werden kann, so dass sich bei Labortieren vor einer Tomografie eine bisher vorgeschriebene Ernährungsumstellung erübrigt.

## Resumen

La comida comercial para ratones está diseñada para ofrecer una dieta completa y rica en nutrientes, y puede contener más de 100 mg/kg de manganeso (mineral esencial).El manganeso actúa como un agente de contraste que acorta el periodo de relajación para T1 y T2, y cuando la comida estándar se hidrata en el tubo gastrointestinal se producen señales brillantes usar escáners de secuencia T1 (T1WI).Debido a la peristalsis, las hiperintensidades gastrointestinales provocan señales temporalmente inestables, lo cual lleva a una reducción de la resolución y una difuminación de las imágenes superiores a las recomendadas. Para evitar el problema, se han documentado varios métodos de modulación del tubo gastrointestinal incluido el uso de limpiado intestinal con laxantes y una modulación dietética. La modulación dietética ha ampliado el uso de un cambio de dieta a largo plazo y biológicamente inocuo. En este estudio, hemos documentado el uso de un alimento sin manganeso disponible comercialmente para mejorar la calidad de las imágenes tomadas del tubo gastrointestinal. Esta comida sin manganeso, aparte del manganeso omitido que está disponible en el agua corriente, es una dieta completa y disponible al instante. Investigamos las intensidades gastrointestinales relacionadas con la dieta y con dependencia del tiempo en T1W1 MRI de TR breve; controlamos la masa corporal, así como el consumo de agua y comida y un análisis bioquímico de sangre estándar después del cambio de dieta; y determinamos la concentración de manganeso en el plasma sanguíneo después de un cambio de 5 días a comida sin manganeso. Demostramos que la comida sin manganeso ofrece un refinamiento en comparación a otras modulaciones del tubo gastrointestinal ya que evita la necesidad de procedimientos invasivos para el vaciado intestinal y puede evitarse ad libitum para que los animales puedan mantenerse sin necesidad de un cambio de dieta prescrito antes de los escáners.

## Introduction

In preclinical magnetic resonance imaging (MRI), the gastrointestinal tract often appears bright when using short-TR, T_1_-weighted imaging (T_1_WI).^1,2^ Although some applications such as bowel-disease diagnosis and small-bowel motility studies benefit from high signal intensities in the gut,^3,4^ this can pose a problem in target identification in other parts of the abdomen.^1^ Peristalsis results in temporally unstable signals, leading to image ghosting and decreased resolution from that prescribed. Therefore, high signal intensities derived from food in the gut are best avoided when performing MRI of the abdomen.

Commercial rodent chow is designed to provide a complete and nutrient-rich diet that, in the European Union, can contain upwards of 100 mg/kg manganese in the form of crystallised manganous oxide powder.^5^ Mn^2+^ is paramagnetic, acts as a relaxation time-shortening contrast agent for both T_1_ and T_2_ and is one of the few ions that can generate similar MRI contrast to gadolinium.^6^ As such, bright signals are produced where this food is hydrated in the gastrointestinal tract. Consequently, an obvious and relatively easy solution to reduce gut hyperintensities might be to remove the added manganese from rodent chow. However, manganese is an essential mineral, necessary for a variety of metabolic functions and, as such, cannot be omitted from the diet altogether.^5^ Blood, serum and plasma levels have been utilised as simple biomarkers for manganese status.^7–10^ Although blood manganese levels are about 10 times higher than plasma levels, whole blood manganese seems to be extremely variable. In contrast, several studies have reported that serum or plasma manganese concentrations are sensitive to dietary intake.^11^ Consequently, nutritional deficiency of manganese may be more readily diagnosed from its concentration in plasma than in whole blood.

Diet represents the major source of manganese. Although manganese intake from drinking water is substantially lower than intake from food, its bioavailability in water may be higher.^12^ In drinking water, manganese is present predominantly in the divalent form Mn(II), that is, the form more likely to be absorbed from the gastrointestinal tract.^2,13^ Manganese is commonly found in groundwater because of the weathering and leaching of manganese-bearing minerals and rocks into the aquifers. So, it might present a good alternative to the manganese additives in rodent chow. In the USA and the UK, the maximum concentration of manganese permitted in drinking water is 50 µg/L (Drinking Water Inspectorate, UK; Environmental Protection Agency, USA).

The confounding effects of gastrointestinal hyperintensities on preclinical MR lymphography and the identification of abdominal lymph nodes have been described previously.^1^ In an effort to improve image quality, the effect of various dietary preparations such as dietary modulation, intestinal cleansing using laxatives and fasting were investigated. Only dietary modulation, in this case by using a potato or sweet potato diet, reduced gastrointestinal signals and image degradation due to motion artefacts enough to facilitate lymph node detection significantly. However, the mice needed to be fed this modified diet for at least 24 hours to clear the gut of the standard food, preventing arbitrary selection of animals for imaging at short notice. Moreover, the effects of prolonged dietary control were not tested, and it is unclear if the modified diet fulfils the nutritional adequacy for rodents, as it is known, for example, that potatoes are lacking in vitamins A and E.^14^

In this study, we report on the use of a commercially available manganese-free chow to improve image quality of the gastrointestinal tract. This chow, apart from the omitted manganese which is available in tap water, is a complete diet and readily available. We investigated the time-dependent, diet-related gastrointestinal intensities on short-TR T_1_WI MRI, and we determined manganese blood plasma levels following a five-day change to manganese-free chow and established that repeated feeding with the manganese-free chow results in the same body-mass gain and the same blood biochemistry and haematocrit levels as for standard chow. We show that the manganese-free chow avoids the need for invasive procedures for invasive gut voiding and can be provided ad libitum so that animals can be maintained with no need for diet change before imaging.

## Materials and methods

### Animals: general information

All animal procedures were performed in accordance with the UK Animals (Scientific Procedures) Act 1986 (PPL 30/3266, PPL 30/3115) and with local Animal Welfare and Ethical Review Body approval. Mice were housed in individual ventilated polycarbonate solid-bottomed cages. Airflow within the cages was on a positive pressure and was controlled electronically by an IVC air handling system (Techniplast, UK). A 12-hour light/dark cycle was implemented, with the ambient air temperature set at 21 ± 2℃ at 55 ± 10% humidity. All animals were provided with a certified rodent diet, tap water ad libitum, bedding and nesting material and cage enrichment. Diets were changed following transfer into clean cages.

Female six- to eight-week old CBA/CaCrl mice (13.9–18.1 g for longitudinal imaging and 15.0–20.0 g for body-mass monitoring) or 12- to 14-week old (19.8–24.3 g; blood plasma) and six- to seven-week old (21–24 g; dynamic contract-enhanced (DCE) MRI) Crl:NU(NCr)-*Foxn1^nu^* mice (*Mus Musculus*; Charles River, UK) were used. Female CBA/CaCrl mice were chosen, as whole-body MRI imaging in our facility is restricted to mice, and the majority of these are female. As this study investigates a general problem associated with diet and not animal model/strain/sex/biology, we chose a generic longevity mouse strain and not a specific model. Six- to eight-week-old mice were selected for longitudinal imaging and body-mass monitoring, as this age range ensured that the mice would fit within the 25-mm ID birdcage coil that was used for whole-body imaging. Stock animals were used to determine blood plasma manganese levels so that we did not have to commission any more mice than strictly necessary. We have no rationale to suppose that basic body weight would alter manganese uptake, and most of the studies performed in our imaging facility are performed in animals within this higher weight range. Crl:NU(NCr)-*Foxn1*^nu^ mice were used to allow for BxPC-3 pancreatic xenografts to grow.

Body-mass monitoring was performed in a total of 40 mice, randomly assigned to two treatment groups and housed in groups of five per cage. To determine blood plasma manganese levels, a total of 10 mice were randomly assigned to two treatment groups, with each group housed in a different cage, and plasma samples were processed blinded to the treatment group.

Longitudinal imaging was performed on another 10 mice that were housed in groups of five per cage. Body weight and visual inspection based on the mouse grimace scale were used to monitor the animals throughout the experiments. Euthanasia, both for welfare reasons and at the end of the study, was performed by cervical dislocation, and death was confirmed by rigor mortis. One mouse from the five used in the five-day imaging study was euthanized following the third anaesthetic session due to a 1.1 g weight loss over 24 hours. Post-mortem examination could not identify any obvious cause for this, although the entire alimentary canal was free of food content.

Anaesthesia was induced and maintained using isoflurane (typically 4% and 1–2%) in room air supplemented with oxygen (80%/20% v/v; flow rate 1 L/min). Inhalable isoflurane anaesthesia was chosen to aid rapid recovery following imaging and because of its quick elimination through the lungs and relative technical ease of administration. Anaesthesia depth was based on respiration rate monitored using a pneumatic cushion, and isoflurane levels were adjusted to maintain a respiration rate of 40–60 breaths per minute. Induction and recovery were performed in a heated unit with a base temperature of around 37℃. Throughout the imaging experiments, mice were maintained at a core temperature of 36℃ using a resistive heater interfaced to a homoeothermic maintenance controller.^15^

### Monitoring of body mass, food and water consumption and blood composition

Forty mice were assigned randomly to two treatment groups, and housed in groups of five per cage. All mice underwent five cycles of food change during which the animals were monitored for body mass, and food and water consumption was estimated. In addition, welfare assessment sheets were completed to assess changes in general appearance, unprovoked and provoked behaviour and respiration. Each cycle started (days 0, 7, 14, 21 and 28) when the mice were weighed and transferred to clean cages; the amount of fresh food and tap water provided was measured. After five days (days 5, 12, 19, 26 and 33), mice were weighed; food chow and water were weighed and replaced with fresh food and tap water. Food and water consumption were estimated as the total mass of food and water taken from the food hopper and water bottle, respectively, and normalised to the consumption per mouse per day. At the end of the fifth cycle, five mice from each treatment group were randomly selected in order to determine standard blood biochemistry (sodium, potassium, chloride, total carbon dioxide, ionised calcium, glucose, urea nitrogen and creatinine; i-STAT Chem8 + cartridge; Abbott, UK) and haematocrit levels in a terminal cardiac blood sample.

Animals in group 1 (*n* = 20) were fed a global 18% protein rodent diet (Teklad 2918 irradiated; Envigo, UK; containing 100 mg/kg manganese in the form of manganous oxide; referred to as standard chow) for five days. On day 5 of each cycle, fresh standard chow and tap water were provided for two more days. Animals in group 2 (*n* = 20) were fed a manganese-deficient rodent diet (TD.140857; Envigo, UK; referred to as manganese-free chow) for five days. On day 5 of each cycle, fresh standard chow and tap water were provided for two days. The manganese-deficient diet was modified from AIN-93 G (TD. 94045), a commonly used complete rodent diet, in order to eliminate the added manganese. This schedule was set within the limits of the experimental licencing, detailed above, whilst the effects of diet change were under investigation.

Variables known to influence body weight were minimised as far as possible: each cage was presented with the same bedding material and cage enrichment; cages were handled only on the days when food was changed; traffic passing the cages was kept to a minimum; and tunnel handling was applied to minimise stress during handling.

### Determination of manganese concentrations in blood plasma and tap water

Following transfer to clean cages, mice were fed either standard chow (*n* = 5) or manganese-free chow (*n* = 5) for five consecutive days.

Five days post diet switch, cardiac blood sampling under terminal anaesthesia was performed using a 1 mL syringe (Braun, VWR, UK) and 23 G needle (Terumo, VWR) pretreated with Heparin (1000 IU/mL; Wockhardt Ltd, UK). Full blood was transferred to heparin-coated vials (item 12947646; Thermo Fisher Scientific, UK) which were gently rotated and immediately placed on ice. All blood samples were spun for 10 minutes at 2000g and at 4℃ (Eppendorf 5417R centrifuge). Plasma was collected, transferred to cryovials (1.5 mL; Thermo Fisher Scientific) and stored at –20℃ until processed.

The preparation of the blood samples for the microwave digestion was performed in a clean room, and all chemicals were acquired from Sigma–Aldrich (UK) unless stated otherwise. Quartz sub-boiled distilled nitric acid (15 M), double deionised water (>18 MΩcm) and 30% hydrogen peroxide (Romil Ltd, UK) were used throughout the procedure. Mouse blood (0.5 mL) was transferred to acid-cleaned XP-1500 Plus (PTFE) vessels, and 4.5 mL distilled nitric acid and 1.5 mL hydrogen peroxide were added. For quality control, a blank sample consisting of only nitric acid and hydrogen peroxide was processed. The samples were pre-digested at room temperature overnight before starting the microwave acid digestion. The samples were digested using the MARS5 microwave apparatus (CEM Corp., UK) by ramping up the temperature stepwise to 210℃ and 250 psi over 60 minutes, and held there for 30 minutes to ensure complete digestion. After cooling, the digested samples were transferred to Savillex Teflon® vials and evaporated until dry at 100℃ on a hotplate. The dried samples were redissolved in 5 mL 2% nitric acid refluxed at 80℃ for two hours, cooled down and transferred to ‘metal-free’ centrifuge tubes (VWR) for manganese analysis.

The manganese concentration of the tap water in our animal unit was determined; water samples were taken immediately and after the tap was run for five minutes. Inductively coupled plasma mass spectrometry (ICP-MS) was used to determine manganese concentration in the blood plasma samples and tap water. The PerkinElmer (USA) NexION 350D ICP-MS, equipped with an Elemental Scientific (Omaha, USA) prepFAST M5 autosampler and autodiluter, was employed. Prior to undertaking the measurements, the instrument was optimised using a set-up solution (containing 1 µg L-1 Be, Li, Mg, Fe, In, Ce, Pb and U) to ensure that the highest sensitivity was obtained (using In at m/z 115 as a proxy), whilst maintaining CeO/Ce (m/z: 156/140) to be <0.025. Tuning of the quadrupole ion deflector lens plate voltages was also undertaken to obtain maximum transmission. Finally, a performance test was undertaken to ensure that the relative standard deviation from five replicate scans was < 3%, in addition to the sensitivities for Be (m/z 9), In (m/z 115) and U (m/z 238) being >2000 cps, 40,000 cps and 30,000 cps, respectively; the ratio of Ce++/Ce+ (m/z 70/140) being < 0.03; and the background (using data from m/z 220) being < 1 cps. The method to measure the samples used the PerkinElmer Syngistix software, which also contained the calibration and timing information for the prepFAST operations. All blanks, standards and samples were analysed using m/z 55 at a dwell time of 25 ms from a series of 60 sweeps and five replicates. The prepFAST diluted the samples by a twofold in-line dilution and also added aliquots of Rh, which were also measured from m/z 103 as an internal standard. Helium was used as a cell gas to perform kinetic energy discrimination in order to reduce polyatomic interferences on the analyte mass. The instrument was calibrated from a diluted stock of a custom blend multi-element standard (produced by CPAchem Ltd and distributed by VWR), which was diluted in-line by the prepFAST to produce a series of calibrants from 0 to 10 ng/g Mn. As quality control, a separate diluted multi-element standard (produced by CPAchem Ltd and distributed by Qmx Laboratories, UK) was also measured to verify the calibration.

### Longitudinal MRI imaging of gastrointestinal hyperintensities and body-mass monitoring

Five CBA mice were imaged longitudinally at 0, 12, 16 and 24 hours following diet change (diet changed at 5:00 am) from standard to manganese-free chow. Five CBA mice were imaged longitudinally at 0, 21, 45, 68, 89 and 117 hours following diet change from standard to manganese-free chow, and 10 further mice were monitored for five days following the same diet change (diet changed at 10:00 am).

MRI was performed on a 7.0 T 210 mm horizontal bore VNMRS preclinical imaging system equipped with 120 mm bore gradient insert (Varian, Inc., USA). RF transmission and reception was performed with a 100 mm long 25 mm ID quadrature birdcage coil (Rapid Biomedical GmbH, Germany). Cardio-respiratory gated steady-state 3D spoiled gradient echo imaging^16^ was performed with TR 1.6 ms, TE 0.7 ms, FOV 108 mm × 27 mm × 27 mm, matrix 256 × 64 × 64, gradient spoiling with 168 mT/m for 0.45 ms in all three axes, RF hard pulse duration 16 μs, FA 5° and RF spoiling. Sixty-four k-space lines were acquired in a centre-out order in a time of 103.4 ms whenever the cardio-respiratory control signal registered an inter breath R-wave. Data from the two heartbeats acquired prior to detection of each breath were reacquired immediately after the same breath. Twenty repeats of the 3D scan were performed. MRI images were processed using ImageJ^17^ and Matlab (Mathworks). Maximum intensity projections of the averaged image of the dynamic time series were created to show the hyperintensities in the complete gastrointestinal tract. In addition, a central slice for each mouse was presented to show the bright intensities in the T_1_-weighted images.

### DCE-MRI in a mouse xenograft model of pancreas cancer

Six BxPC-3 pancreatic xenograft bearing Crl:NU(NCr)-*Foxn1*^nu^ mice were imaged using DCE-MRI to demonstrate the application of the manganese-free chow. Tumours were generated through subcutaneous inoculation of 1 × 10^6^ BxPC-3 cells in 1:1 supplement-free RPMI media (31870-025; Thermo Fisher Scientific) and Matrigel (356234; Corning, UK). When tumours reached 50 mm^3^, DCE-MRI was initiated to characterise the tumour model in terms of tumour vascularisation and perfusion. The foodstuff was changed to the manganese-free chow 48–60 hours prior to imaging.

A 32-mm ID quadrature birdcage coil (Rapid Biomedical GmbH) was used, together with a cardio-respiratory gated 3D spoiled gradient echo scan; TR 1.6 ms, TE 0.632 ms, FOV 64 mm × 32 mm × 32 mm, matrix 128 × 64 × 64, gradient spoiling with 159 mT/m for 0.432 ms in all three axes, RF hard pulse duration 16 μs, FA 5° and RF spoiling. Data were acquired in blocks of 64 k-space lines and the two data blocks acquired prior to detection of each breath were reacquired immediately after the same breath.^16^ Fifty repeats of the 3D scan were performed with 30 µL of a gadolinium contrast agent (Omniscan, GE Healthcare) infused via a tail-vein cannula over five seconds, starting at the beginning of frame 11/50.

### Statistics

Statistical analysis was performed using GraphPad Prism v7.04 (GraphPad Software, USA). The Mann–Whitney non-parametric test was used to calculate significance of difference for manganese concentration in plasma and for blood analyte concentrations following diet change. Gaussian distribution was verified using the Shapiro–Wilk normality test. All plasma and blood biochemistry data were obtained in quintuple independent replicates, and results are reported and graphed as the mean ± *SD*. Two-way analysis of variance tests followed by Sidak’s multiple comparisons test were used to analyse the grouped data obtained from the body-mass monitoring experiment.

## Results

### General welfare

None of the animals showed any changes in general appearance, unprovoked and provoked behaviour or respiration, as assessed by the welfare assessment sheets. No loss of body weight or visual deterioration of general health based on the mouse grimace scale was observed.

### Determination of manganese concentrations in plasma and tap water

**Figure 1.**
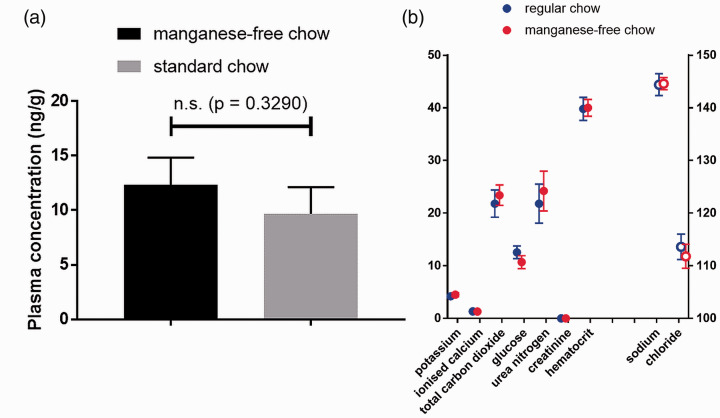
(a) Blood plasma manganese and (b) blood biochemical analyte levels for CBA/Crl mice (*n* = 5/group) five days post diet switch from a standard global 18% protein rodent diet to a manganese-deficient diet. The sodium and chloride electrolyte concentrations are depicted on the right *y*-axis for clarity. The units for sodium, potassium, chloride, ionised calcium and total carbon dioxide are mmol/L. The units for glucose, creatinine and urea nitrogen are mg/dL. Haematocrit is expressed as % packed cell volume.

The tap water in our animal unit contains 0.56–0.65 µg/L manganese, the concentration being dependent on how long the tap was running before taking the sample. Analysis of blood plasma manganese levels showed no significant difference between the standard chow group and the manganese-free diet group five days post food change. The results are presented in Figure 1(a).

### Standard blood analysis

Moreover, standard blood analyte concentrations, as shown in Figure 1(b), were not significantly different between mice fed regular chow or manganese-free chow; *p*-values of 0.8548, 0.1181, 0.2595, 0.2063, 0.2222, 0.0566, 0.3394 and 0.8718 were obtained for sodium, potassium, chloride, total carbon dioxide, ionised calcium, glucose, urea nitrogen and haematocrit, respectively (creatinine levels were below the detection limit).

### Body-mass monitoring

**Figure 2.**
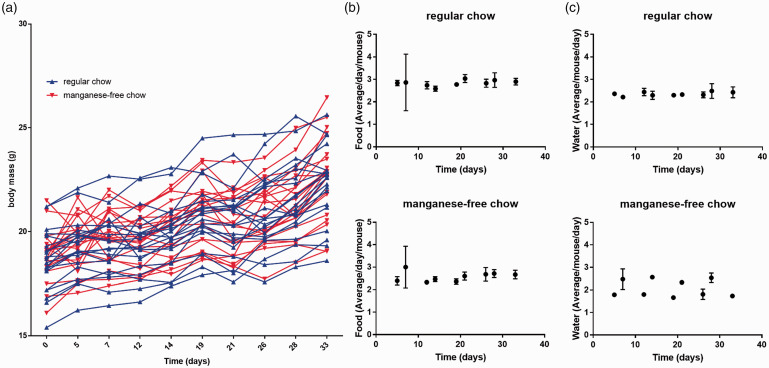
Body-mass monitoring and food and water consumption for mice fed on regular or manganese-free chow. (a) Body-mass monitoring for each individual mouse over a five-week period. Blue and red triangles depict mice being fed regular and manganese-free chow, respectively. (b) Food and (c) water consumption normalised per experimental day and per animal for mice fed regular (top) and manganese-free (bottom) chow.

The results for body-mass monitoring during five cycles of food change of 40 mice are presented in Figure 2(a). No significant difference in body mass was detected between mice fed regular chow or manganese-free chow for either any single time point (*p* = 0.9891– > 0.9999) or over the five cycles combined (*p* = 0.7273), although both groups of mice increased in body weight equivalently.

### Food and water consumption

The results for the estimates for food and water consumption, normalised per experimental day and per animal, are presented in Figure 2(b) and (c), respectively. The mice presented with the regular chow consumed as much food as those presented with the manganese-free chow. Again, this was true for each individual time point (*p* = 0.7025–0.9998) and for the five cycles combined (*p* = 0.0595). Even when the manganese-free chow group was presented with regular chow for two days, food consumption did not change significantly compared to those presented with regular chow throughout the experiment (*p* = 0.3535). Water consumption, however, was different. The manganese-free chow mice showed a lower water intake over the five-day sub-cycle (when they received manganese-free chow) compared to those fed regular chow (*p*-values for all five cycles = 0.0007). When mice in the manganese-free chow group were presented with regular chow for two days, water consumption was not significantly different from that of the regular chow mice (*p* = 0.3210– > 0.9999). One data point for water consumption for group 2 was lost due to a profound water leak, but there is no indication that this changed the statistical analysis.

### Longitudinal MRI imaging of gastrointestinal hyperintensities and body-mass monitoring

**Figure 3.**
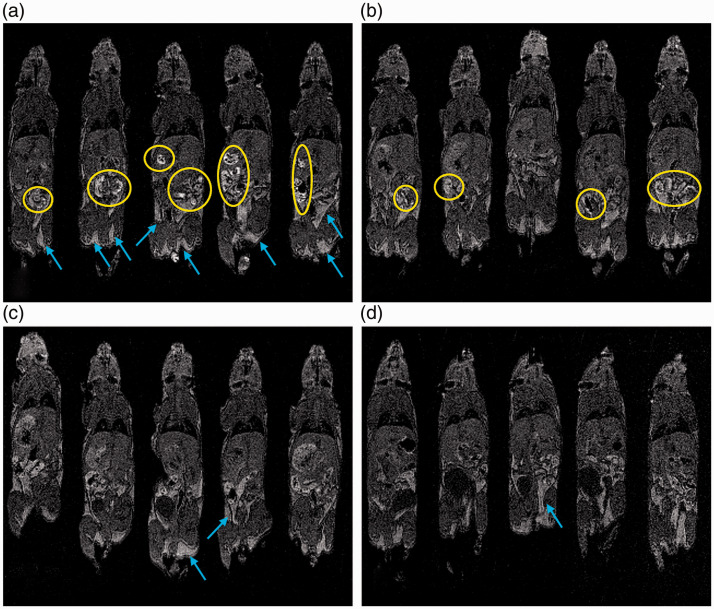
Single-frame anatomical magnetic resonance imaging (MRI) of the gastrointestinal tract over time. Each panel depicts a representative coronal slice for five different mice imaged at (a) 0 hours, (b) 12 hours, (c) 16 hours and (d) 24 hours post diet switch from standard to manganese-free chow. Food-related gut hyperintensities are highlighted in yellow. Residual bright signals at later time points are primarily derived from fat depots around the body of which examples are highlighted by blue arrows.

**Figure 4.**
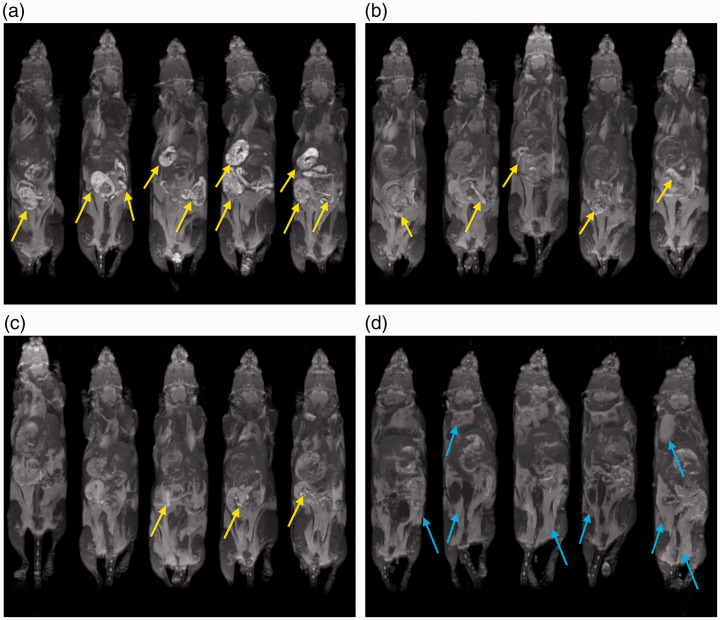
Evaluation of the change in gastrointestinal hyperintensities over time. Each panel depicts the maximum intensity projection for five different mice imaged at (a) 0 hours, (b) 12 hours, (c) 16 hours and (d) 24 hours post diet switch from standard to manganese-free chow. Images acquired at later time points appear similar to the 24-hour time point. Examples of food-related hyperintenisties and fat depots are highlighted in yellow and blue, respectively.

Single slices through the gastrointestinal tract and maximum intensity projections for each mouse at each time point are shown in Figures 3 and 4, respectively. The single-slice anatomical images highlight the signal intensity of the gastrointestinal contents and demonstrate how it interferes at a local level. However, it is difficult to represent a 3D volume faithfully in such a 2D representation. So, maximum intensity projections are presented to show the long-term, time-dependent spatial distribution of food in the gut.^1^

Images produced after feeding with standard chow showed multiple regions of hyperintensity that reduced in number and intensity over time after switching the same animals to the manganese-free chow (Figure 3). At 24 hours, but not before, the hyperintensities in the gastrointestinal tract were markedly reduced and barely noticeable upon visual inspection. This was confirmed by the maximum intensity projections (Figure 4). At later time points, the anatomical images and the MIP images look similar to those presented for the 24-hour time point (data not shown, as the images do not show the food-related hyperintensities).

### DCE-MRI in a mouse xenograft model of pancreas cancer

**Figure 5.**
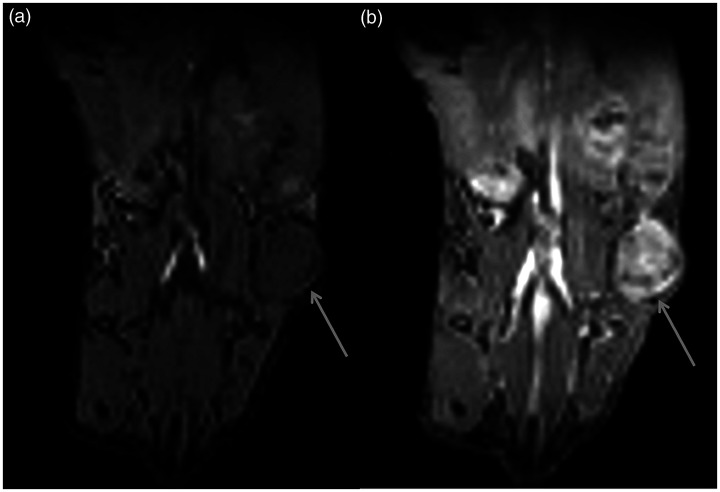
Manganese-free chow diet applied to dynamic contract-enhanced MRI of a mouse xenograft model of pancreatic cancer. A slice through a tumour (arrow) with the gut immediately adjacent is shown (a) before and (b) after gadolinium injection. Major blood vessels can also be observed.

The manganese-free chow approach was applied to a study investigating the perfusion characteristics of a pancreatic xenograft tumour model. Six animals were imaged, of which a representative image, showing a slice through a tumour with the guts immediately adjacent, is shown in Figure 5. DCE-MRI clearly identified the tumour following gadolinium injection, and heterogeneity within the tumour can be observed. Although not all hyperintensities from the gut were cleared over 48–60 hours following diet change, Figure 5 shows that the remaining level of hyperintensities did not affect the dynamically acquired data.

## Discussion

A nutrient-rich manganese-deficient diet was produced on our request from a simple modification of a standard animal foodstuff. The modified diet is commercially available, and apart from its deficiency in manganese, it is nutritionally complete. We have shown that use of this manganese-free diet reduces the intensity of food in T_1_WI of the gastrointestinal tract over time, whilst the plasma manganese levels remain intact. Since the demonstration of its use, the manganese-free chow has been adopted by our user group to reduce gastrointestinal hyperintensities in DCE-MRI and other MRI. Although the hyperintensities do not always clear completely from the gut, they are markedly reduced and do not affect the dynamically acquired data, and as such this avoids propagation of errors derived from food-related image artefacts. Longer-term use of the diet will ensure the gut is completely clear.

Body weight increased equivalently between the two groups of mice over the five-week period. Food consumption, estimated as the mass of food removed from the hopper, was also equivalent. Water intake, estimated from the mass of water removed from the bottle, showed a significantly lower consumption during feeding with manganese-free chow. The reason for this is not clear, but as body weights increased, food intake was maintained equivalently between groups, welfare assessments and blood biochemistry analysis did not detect anything abnormal and water was available ad libitum, there was no indication of dehydration. It has previously been shown that water intake is changed under conditions of differing purified foodstuff,^18^ and this, in conjunction with the experimental findings described above, suggests that this altered water intake is innocuous.

Dietary modulation for reduced hyperintensities in the gastrointestinal tract of rodents during T_1_WI, has been described previously,^1^ and here it is extended to use of a biologically innocuous long-term change of diet. This enables non-selective preparation of animals for imaging, providing major refinements for animal handling and a greatly streamlined workflow. The manganese-free diet avoids the need for invasive procedures for gut voiding and can be provided ad libitum to all littermates. Since animals can be maintained with no need for diet change before imaging, as no preparation is required, animals may be selected for imaging without notice using scientifically rational criteria, and do not need to be removed from their home cages and cage mates whilst standard chow is eliminated. Moreover, as long as the animals are given free access to tap water, the nutritional requirements are met, as indicated by the intact plasma manganese levels. This is in contrast to the previously tested potato or sweet potato diet which is not nutritionally balanced and where rodents would quickly become deficient, at minimum, in vitamins A and E.^14^ As a result, such dietary change is a short-term solution.

When feeding a manganese-free chow whilst retaining normal bioavailability of manganese, delivery of manganese needs to be maintained. The manganese blood plasma results show that this was achieved over five days. The concentration of manganese found in the tap water in our animal facility is in line with those reported by the Drinking Water Inspectorate. The 1st and 99th percentiles, representing a minimum and maximum, for the concentration of manganese in drinking water supplied by Thames Water in 2017 were < 0.8 µg/L and 2.2 µg/L, respectively (www.dwi.gov.uk). In the event that further supplementary manganese is required, it could be added to the drinking water without causing the food-related hyperintensities described.

Longitudinal imaging of the CBA mouse abdomen revealed a time period of more than 16 hours but less than 24 hours for the hyperintensities caused by the crystallised manganous oxide powder present in standard chow to clear. This gastrointestinal transit time of the food is longer than was anticipated from the literature.^19,20^ This transit time is a problem for a modified diet such as the potato diet, as the diet needs to be freshly prepared and fed exclusively at a set time, for example 24 hours prior to imaging, it is not nutritionally complete and it requires that animals be segregated prior to imaging. It also means that coprophagia, which helps rodents obtain vitamins, minerals and other nutrients, must be avoided, as most ingested manganese will not be absorbed by the gut and will appear in the faeces, facilitating the extended cycle.^21,22^ Some hyperintensities were seen in the DCE-MRI study of Crl:NU(NCr)-*Foxn1*^nu^ mice even at 48–60 hours post food change. It is not clear if this is idiopathic, pathologic or strain-related, but it may indicate that diet changes may need to be long term for optimised imaging. Whilst a potato diet can help with scheduled procedures, it precludes the use of rational selection of animals for imaging, as may be required by the time-dependent biology in question, and may hinder the utilisation of instrument time where it becomes unexpectedly available. When the diet switch is made at short notice, then waiting for extended periods for the gut contents to change might be long enough to miss the window of opportunity for imaging and/or treatment, or for animals to reach their humane end point. As such, we envision that animals could be kept on a diet such as that described in this paper from the start until the humane end point of the experiment. Whilst there is no need to maintain animals on this diet at a time when imaging would not be anticipated, it is a nutritionally complete diet when manganese is available in the drinking water.

Utilisation of this manganese-free chow requires that sufficient Mn^2+^ be provided to maintain bioavailability of this ion. True manganese deficiency has been shown to lead to skeletal malformations, infertility of the offspring resulting from testicular degeneration and problems with suckling the young. However, even small amounts (50 ppm) were sufficient to ameliorate the effects.^23^ It is quite possible to provide further supplementary manganese via occasional feeding with, for example, pineapple or blueberry juice (https://ndb.nal.usda.gov/ndb/nutrients/), which could ensure adequacy of bioavailability, thus removing the need for regular monitoring of the water content of the drinking water.

In conclusion, gastrointestinal hyperintensities in T_1_WI due to manganese ingestion can be avoided using a simple change to a manganese-free diet. The level of manganese in the food can be reduced to zero in cases where the dietary need is satisfied by trace levels of manganese in the drinking water.
